# Exploring Differences Between Adolescents and Adults With Perinatal Depression—Data From the Expanding Care for Perinatal Women With Depression Trial in Nigeria

**DOI:** 10.3389/fpsyt.2019.00761

**Published:** 2019-10-24

**Authors:** Bibilola D. Oladeji, Toyin Bello, Lola Kola, Ricardo Araya, Phyllis Zelkowitz, Oye Gureje

**Affiliations:** ^1^Department of Psychiatry, College of Medicine, University of Ibadan, Ibadan, Nigeria; ^2^Department of Health Services and Population Research, King’s College London, London, United Kingdom; ^3^Division of Social and Transcultural Psychiatry, McGill University, Montréal, QC, Canada; ^4^Department of Psychiatry, Jewish General Hospital, Montréal, QC, Canada

**Keywords:** adolescents, women, perinatal depression, primary health care, low- and middle-income countries

## Abstract

**Background:** Depression is common among women in the perinatal period. Although pregnancy and motherhood among adolescents are global public health issues, little is known about how adolescents differ from adults in the occurrence and correlates of perinatal depression.

**Methods:** Data were derived from a cluster randomized controlled trial of psychosocial interventions for perinatal depression in primary maternal care in Nigeria (the Expanding Care for Perinatal Women with Depression trial). Adolescents and adult participants recruited during pregnancy and followed up till 6-month postpartum were compared: proportions with depression [screening positive to depression on the Edinburgh Postnatal Depression Scale (score ≥ 12) and meeting the Diagnostic and Statistical Manual of Mental Disorders, Fourth Edition, criteria using the short form of the Composite International Diagnostic Interview]; adjustment and attitude to pregnancy and motherhood (using the Maternal Adjustment and Maternal Attitudes scale); and parenting skills (measured on Infant–Toddler version of the Home Inventory for Measurement of the Environment). Infant and fetal growth were assessed by measures of weight and head circumference at birth and upper mid-upper arm circumference (MUAC) at 6 months.

**Results:** Of 8,580 adults screened, 6.9% had major depression compared with 17.7% of 772 screened adolescents (p < 0.001). Adolescents had significantly poorer adjustment and attitudes to pregnancy, lower mean fetal gestational age at birth, and a smaller mean baby’s birth weight. At 6-month postpartum, there were no significant differences in the rates of remission from depression between adolescent and adult women (Edinburgh Postnatal Depression Scale score <6). Adolescent mothers continued to have poorer maternal attitudes and parenting skills indicated by significantly lower scores on the Infant–Toddler version of the Home Inventory for Measurement of the Environment responsivity and involvement subscales. Infants of adolescent mothers had a higher rate of undernutrition (defined as MUAC < 12.5 cm) compared with those of adult mothers: 14.8 and 6.3%, respectively (p = 0.008), with the mean MUAC remaining significantly lower for infants of adolescent mothers after adjusting for their lower birth weight (p = 0.04).

**Conclusion:** Perinatal depression is more common and is associated with poorer maternal attitudes and parenting skills in adolescents compared with those in adults. Evidence from this exploratory study suggests that in improving outcomes in infants of adolescent mothers with perinatal depression, depression treatment may need to be supplemented with specific approaches to improve parenting skills.

## Introduction

The World Health Organization (WHO) estimates that despite the declining global rates of adolescent pregnancy, up to 11% of all births worldwide are still to girls aged between 15 and 19 years (1). The global rate for adolescent pregnancy in the 2015 World Health Statistics is about 44 per 1,000 girls between the ages of 15–19 years with a range of 1–201 across countries. The highest rates are in countries in sub-Saharan Africa; Nigeria, for example, has a birth rate of 109 per 1,000 adolescents in 2015 down from a peak of 172/1,000 in 1977 (2). Adolescent pregnancy often presents a unique set of challenges and risk factors compared with pregnancy in adulthood. In addition to contending with normal demands of navigating the developmental tasks of adolescence, pregnant adolescents have to adapt to the responsibilities and demands associated with becoming a parent. Adolescent pregnancy is associated with adverse birth outcomes including elevated risks of obstetrical complications, lower birth weight babies, and maternal mortality (3–5). In addition, adolescent mothers who face poor social and economic conditions and prospects are more likely to experience parenting difficulties and further pregnancies in adolescence (6, 7).

An important associated morbidity of adolescent pregnancy is mental illness (8). Compared with adult women, pregnant adolescents are at a significantly increased risk for common mental disorders (9). For example, the risk for perinatal depression, a condition that has received the most attention in the literature, is double in adolescents relative to older women (10, 11). The prevalence rates for perinatal depression in adolescents are estimated to be between 16 and 44% compared with 5–20% in adult women (7, 9, 12). The risk factors for perinatal depression in adolescents are largely similar to those in adults and include low socioeconomic status and low perceived social support (9, 10).

Similar to findings in adult women, perinatal depression in adolescent mothers is associated with negative birth and infant outcomes. Perinatal depression in adolescent mothers increases the risk for small-for-gestational-age babies and preterm delivery (13). In addition and more specifically, adolescent mothers with depression have poor interactions with their babies and are more likely to use aggressive parenting behaviors with their children (14). Children of adolescent mothers are more likely to develop preschool problem behaviors, have delays in cognitive development, have higher levels of psychopathology, poorer school performance, and are at higher risk of also becoming teenage parents (9, 15).

Till date, there is limited empirical information about effective interventions for prevention and treatment of perinatal depression in adolescents. In a systematic review of the literature conducted in 2014, Lieberman and colleagues were able to identify only two treatment studies (16). One study evaluated the effectiveness of group interpersonal therapy among 11 pregnant girls (mean age 16.5 years) with Diagnostic and Statistical Manual of Mental Disorders, Fourth Edition (DSM-IV) major depression over a 12-week period (17). The other study assessed the impact of a telephone-based depression collaborative care program consisting of motivational interviewing and psychoeducation over 6 months among 97 teenage mothers (mean age 16.4 years) (18). Both studies reported positive outcomes in the adolescents following treatment. Neither of the studies examined impact of treatment on the infants and neither had a control group nor used a randomized controlled design.

Most of the previous studies on risks, consequences, and treatment of perinatal depression in adolescents have come from high-income countries with little available information from low- and middle-income countries where, as previously shown, there is indeed a much higher rate of adolescent pregnancy. More generally, the paucity of rigorously conducted studies on treatment of adolescents with perinatal depression may reflect the difficulties of engaging with and conducting research on this vulnerable group where ethical issues relating to research participation are likely to constitute added disincentive for researchers. Specifically, an important gap in the global literature is information about whether there are unique and peculiar features that set adolescents with perinatal depression apart from adults with the same condition or that may affect their response to treatment. Identifying such unique features will be a first and vital step in designing age-appropriate interventions for this vulnerable group of mothers.

In this exploratory study, we used data from a randomized controlled study conducted in primary maternal care clinics in Nigeria to compare adult and adolescent participants with regard to the occurrence as well as maternal and infant correlates of perinatal depression both during pregnancy and 6-month postpartum.

## Materials and Methods

The data for this report are from the Expanding Care for Perinatal Women with Depression (EXPONATE) trial. This two-arm parallel cluster randomized control trial of psychosocial interventions for perinatal depression in primary health care was conducted in Ibadan, Southwest Nigeria between June 18, 2013 and December 11, 2015. A full description of the study protocol as well as the results of the trial has been published (19, 20), and only a summary of the methodology relevant to the current study will be described here. The unit of randomization was primary maternal care clinics in the study area, while the unit of analysis was individual women participants. Twenty-nine eligible and consenting primary maternal and childcare centers were randomized into either the stepped-care structured psychosocial intervention (high-intensity) arm (15 clinics) and enhanced usual-care (comparison or low-intensity) arm (14 clinics). Eligible clinics were those with a capacity to offer antenatal, delivery, and postnatal services.

Consecutive pregnant women registering for antenatal care at the participating clinics were approached for screening and assessed for eligibility while waiting to be seen by the primary health care worker (PHCW). Screening was conducted by trained research staff using the Edinburgh Postnatal Depression Scale (EPDS). Eligible participants were those with an EPDS score of 12 or more, aged between 16 and 45 years, had fetal gestational age between 16 and 28 weeks, were likely to remain in the area of the study for the duration of follow-up, and provided written informed consent. Women who did not meet diagnostic criteria for DSM-IV major depression, following assessment with the short form of the Composite International Diagnostic Interview, or had a history of bipolar affective disorder or psychotic disorder and those who were actively suicidal were excluded. Participants who consented to the study were offered either a low- or high-intensity psychosocial treatment delivered by primary maternal care providers in either of the two arms of the study. Participants in the high-intensity intervention arm had the EPDS readministered to them by the PHCW during their routine postnatal visits 6 weeks after childbirth to determine the number of additional postnatal intervention sessions.

Standard care in the randomized clinics were delivered by PHCW who had, prior to the trial, received training on the identification and treatment of depression based on the specifications of the WHO Mental Health Gap Action Intervention Guide (mhGAP-IG). In addition to the mhGAP-IG training, providers in the high-intensity treatment arm were trained to deliver a manualized structured stepped-care psychosocial intervention package (20). The package included psychoeducation, activity scheduling (behavioral activation), and a locally adapted form of problem solving treatment delivered in eight prenatal sessions with additional four to eight supplementary postnatal sessions determined by level of remission of patient’s depression symptoms as determined by their EPDS score (21).

Participants in the low-intensity treatment arm were provided with care as usual, that is, unstructured psychosocial interventions based on treatment specification for perinatal depression in the mhGAP-IG consisting of psychoeducation, addressing current psychosocial stressors, and reactivation of social network with no prespecified number of sessions.

The EXPONATE trial from which the data for this study were derived was approved by the University of Ibadan/University College Hospital Ibadan Ethical Review Committee.

### Measures

The primary outcome for the trial was depression remission at 6-month postpartum, defined as EPDS score of less than six. The EPDS is one of the most widely used screening instruments for assessing symptoms of perinatal depression. The EPDS has been used and validated in the several low- and middle-income countries including Nigeria (22).

At baseline, following enrollment, participants were assessed for disability, measured with the WHO Disability Assessment Schedule (23), experience of stigma using the 12-item Discrimination and Stigma Scale (24), and for adjustment and attitudes to pregnancy, with the Maternal Adjustment and Maternal Attitudes (MAMA) questionnaire. These tools, including the postnatal version of MAMA, were readministered at 6-month postnatal, which was the primary outcome point for the trial. Also, at 6-month postnatal, the Infant and Toddler version of the Home Inventory for Measurement of the Environment (IT-HOME) was used to assess the extent to which the infant was receiving adequate home and parental nurturing.

The MAMA is a 60-item questionnaire designed to assess maternal adjustment and attitudes during pregnancy and after delivery. It has demonstrated good acceptability to women and good reliability in the United Kingdom (25) and Portugal (26). For this study, we used the 12 items on the attitude to pregnancy and baby subscale of the MAMA, which includes questions such as “have you been worrying you might not be a good mother?” and “have you been looking forward to caring for your baby’s needs?” Each item is scored on a four-point Likert scale (with negatively worded items reverse scored). In our scoring and analysis, higher scores denoted poorer maternal attitudes. The scale showed good inter-rater reliability (alpha coefficient of 0.7).

The HOME is designed to provide systematic measurement of the family environment. The HOME inventory has different versions designed to explore the home environment at different stages of child development; for this study, we used the IT-HOME. Some items on the HOME are scored based on information provided by the parents and others by direct observation by the interviewer (27). The full scale consists of 45 items, which are scored either yes (1) or no (0) according to the manualized description of each item as adapted for the local context. The scale is divided into six subscales—1) responsivity (a measure of the extent to which the parent responds to the child’s behavior); 2) organization, 3) involvement, and 4) acceptance (assess parental acceptance of less-than-optimal behavior from the child and the avoidance of undue restriction and punishment); and 5) learning materials, and 6) variety.

We followed standard procedures for the cultural adaptation of psychological instruments used in this study (MAMA, HOME, and EPDS) (28, 29). This process consisted of translation and back translation of the items in the tools by a panel of bilingual experts, followed by a review of the items for cultural relevance by a panel of experts (which included persons with professional experience in childcare—a sociologist, social worker, primary care providers, as well as mothers with infants). The study research staff conducting the outcome assessments were then trained, following which reliability exercises were conducted for each tool. For the HOME, in view of the salience of interviewer observation in addition to respondents’ reports, each of the research workers conducted two video recorded assessments that were used for further training and to standardize the administration and scoring of each of the item on the HOME record form. After this standardization process, each conducted two further assessments that were similarly video recorded, and the recordings were used to assess interrater reliability. The instrument showed good inter-rater reliability (alpha coefficient was 0.94 for the full-scale measure on the IT-HOME).

Other outcome measures assessed include birth outcomes (mode of delivery, live births, gestational age at birth, and birth weight), infant growth [mid-upper arm circumference (MUAC) at 6 months], and infant nutrition (practice of exclusive breast feeding for the first 6 months of life). Infant under nutrition was defined according to WHO standards as MUAC < 12.5 cm (30, 31).

Assessments were conducted at baseline, 2 months after baseline, at birth, and 3- and 6-month postpartum in the participants homes by six trained research assistants who were not involved with collecting data in the clinics and were blinded to the arm of the study that the participants were recruited into.

### Statistical Analysis

Data that were saved and uploaded to a secure server and exported into the statistical software were collected using android tablet computers. The data were cleaned and analyzed using STATA (STATA/SE 13.1) software. Student t-tests and ANOVA were used to assess the differences in mean scores between perinatal adolescents and adults on continuous variables such as gestational age, EPDS scores, education, stigma, and disability scores. While categorical variables were compared using the chi-square test. We adjusted the effect of the lower birth weight of adolescent mothers on MUAC at 6 months by entering both variables simultaneously in linear regression models.

## Results

**Figure 1:**
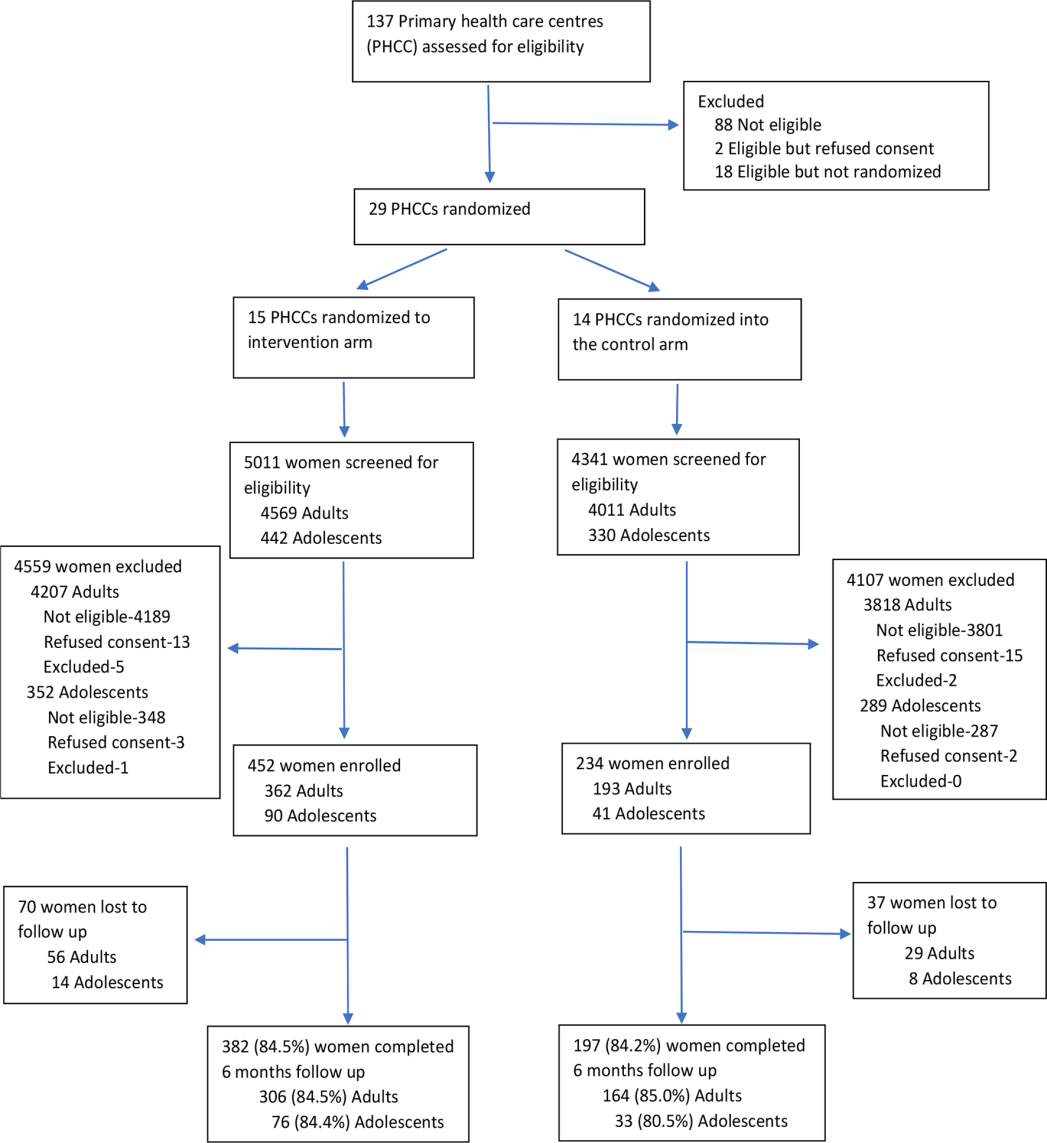
Flow diagram for adolescent and adult mothers.

A total of 9,352 pregnant women were screened with the EPDS; 772 (8.3%) of these were adolescents, aged 19 years and under. Adolescents constituted 20% of the 686 women recruited into the trial (**Figure 1**). We successfully conducted 6-month postdelivery assessments on 579 (84.5%) of the participants; these included 109 adolescents (83.2% of the enrolled adolescents).

The mean age of the adolescents was 17.8 (SD 1.23) years compared with 26.3 (SD 5.07) years for the adults (p < 0.001); 86.8% of the adults were either married or cohabiting with a partner, while for the adolescents, 53.4% reported being married or cohabiting (p < 0.001). The gestational age of mothers at enrollment was similar for adolescents and adult women, at mean 21.3 (SD 3.6) weeks and 22.0 (SD 3.7) weeks, respectively.

Of the 8,580 adult women screened, 590 (6.9%) scored 12 or more on the EPDS and met the DSM-IV criteria for depression. On the other hand, of the 772 screened adolescents, 137 (17.7%) had depression (chi-square = 116.721, p < 0.001). Depression severity at baseline as determined by the mean EPDS score for adolescents (14.9, SD 2.6) was not significantly different from that of the adult population (14.4, SD 2.7) (p = 0.08). At baseline, the adolescents had poorer adjustment and attitudes to pregnancy than adults as indicated by significantly higher scores on the MAMA questionnaire, but the two groups were not significantly different on other parameters (**Table 1**).

**Table 1:** Baseline demographic and clinical variables.

Continuous	High-Intensity Treatment Arm			Low-Intensity Treatment Arm			Total		
	Adolescent mean (SD)	Adult mean (SD)	p-value	Adolescent mean (SD)	Adult mean (SD)	p-value	Adolescent mean (SD)	Adult mean (SD)	p-value
**Age**	17.92 (1.26)	26.19 (5.01)	<0.001	17.63 (1.16)	26.48 (5.18)	< 0.001	17.83 (1.23)	26.29 (5.07)	<0.001
**Education**	10.20 (2.10)	10.71 (3.14)	0.188	9.94 (2.38)	10.56 (3.37)	0.301	10.12 (2.19	10.66 (3.22)	0.096
**Gestational age (weeks)**	21.3 (3.6)	22.0 (3.7)	0.129	22.2 (4.3)	22.6 (4.0)	0.503	21.6 (3.8)	22.2 (3.8)	0.102
**EPDS**	14.70 (2.59)	14.25 (2.71)	0.152	15.27 (2.50)	14.77 (2.58)	0.261	14.88 (2.57)	14.43 (2.67)	0.082
**WHODAS**	18.62 (5.41)	18.06 (4.76)	0.328	19.17 (6.29)	19.53 (6.37)	0.744	18.79 (5.68)	18.57 (5.41)	0.673
**DISC**	15.97 (4.28)	16.69 (4.83)	0.196	16.29 (4.55)	17.55 (5.25)	0.154	16.07 (4.35)	16.99 (4.99)	0.052
**MAMAS**	21.42 (5.30)	19.52 (4.68)	0.001	22.76 (5.85)	20.20 (4.77)	0.003	21.84 (5.49)	19.76 (4.72)	<0.001

EPDS, Edinburg Postnatal Depression Scale; WHODAS, WHO Disability Assessment Schedule; DISC, 12-item Discrimination and Stigma Scale; MAMAS, Maternal Adjustment and Maternal Attitudes Questionnaire (higher scores indicate poorer attitudes); Low-Intensity Treatment Arm, Interventions consist of basic psychosocial interventions specified in the WHO Mental Health Treatment Gap Action Programme Intervention Guide (mhGAP-IG); High-Intensity Treatment Arm, Interventions consisting of the mhGAP-IG interventions, activity scheduling and problem solving treatment.

Even though mode of delivery was similar between the two groups (normal delivery in 98.3% adolescents and 96.8% adults), adolescent mothers delivered at an earlier gestational age and had significantly smaller babies at birth (mean weight: 2.9 versus 3.0 kg, p = 0.002) (**Table 2**).

**Table 2:** Birth outcomes

	Adolescents	Adults	P value
Mode of delivery			
Normal delivery, n (%)	114 (98.3)	489 (96.8)	0.403
Others (Caesarean section or instrumental delivery), n (%)	2 (1.7)	16 (3.2)	
Gestational age at birth, mean (SD)	35.8 (4.1)	37.0 (3.4)	0.001
Birth weight in kilograms, mean (SD)	2.9 (0.4)	3.0 (0.4)	0.002
Head circumference in centimeters, mean (SD)	32.8 (6.5)	33.4 (5.4)	0.318

In the intervention arm, fewer adolescents completed the required eight sessions prenatally compared with adult women (53.8% compared with 63.2%), but this difference was not statistically significant (p = 0.120).

At 6 months after childbirth, depression remission (EPDS < 6) was similar across both arms: 66% in the control arm and 70% in the intervention arm (odds ratio: 1.3; 95% confidence interval: 0.8, 2.0; p = 0.34) (20). In this regard, adolescent mothers with depression were as likely to have experienced remission as the adult women, 70.6 and 68.7%, respectively (odds ratio: 1.09; 95% confidence interval: 0.69, 1.73).

On the other hand, and compared with adult mothers, adolescent mothers continued to have poorer attitudes and adjustment to motherhood at 6-month postnatal follow-up. Parenting skills were also poorer in the adolescent mothers as evidenced by their significantly lower overall scores on the IT-HOME, as well as on the subscale scores of responsivity and involvement (**Table 3**).

**Table 3:** Six month outcome.

	High-Intensity Treatment Arm			Low-Intensity Treatment Arm			Total		
	Adolescents	Adults		Adolescents	Adults		Adolescents	Adults	
6months Secondary outcomes	mean (SD)	mean (SD)	p-value	mean (SD)	mean (SD)	p-value	mean (SD)	mean (SD)	p-value
WHODAS	13.74 (3.36)	13.68 (2.99)	0.891	13.94 (3.77)	14.43 (3.60)	0.480	13.80 (3.47)	13.94 (3.23)	0.679
DISC	13.76 (3.54)	14.24 (3.92)	0.333	14.48 (5.50)	14.87 (4.38)	0.663	13.98 (4.21)	14.46 (4.09)	0.275
MAMAS	21.10 (5.23)	19.55 (3.86)	**0.005**	22.40 (6.43)	20.25 (4.75)	**0.035**	21.48 (5.60)	19.79 (4.19)	**0.001**
**IT-HOME**									
Responsivity	8.04 (1.78)	8.57 (1.79)	**0.025**	7.87 (2.46)	8.56 (1.72)	0.065	7.99 (1.99)	8.57 (1.76)	**0.004**
Organization	4.66 (1.20)	4.95 (0.98)	**0.032**	4.86 (1.13)	4.83 (1.11)	0.884	4.72 (1.18)	4.91 (1.03)	0.101
Involvement	2.89 (0.92)	3.15 (0.95)	**0.037**	3.07 (1.14)	3.24 (1.03)	0.417	2.94 (0.99)	3.18 (0.98)	0.027
Acceptance	6.01 (1.27)	5.94 (1.20)	0.632	5.80 (1.37)	5.85 (1.22)	0.848	5.95 (1.30)	5.91 (1.20)	0.738
Total score	25.90 (4.37)	27.33 (4.56)	**0.018**	25.72 (5.92)	27.05 (4.51)	0.172	25.85 (4.84)	27.23 (4.54)	0.007
Infant	14.3 (1.5)	14.7 (1.6)	0.126	13.9 (1.7)	14.8 (1.7)	**0.017**	14.2 (1.5)	14.7 (1.6)	**0.008**
MUAC [cm]									

WHODAS, WHO Disability Assessment Schedule.

DISC, 12-item Discrimination and Stigma Scale.

MAMAS, Maternal Adjustment and Maternal Attitudes Questionnaire (higher scores indicate poorer attitudes).

IT-HOME, Infant Toddler version of the Home Inventory for Measurement of the Environment.

Low-Intensity Treatment Arm, Interventions consist of basic psychosocial interventions specified in the WHO.

Mental Health Treatment Gap Action Programme Intervention Guide (mhGAP-IG).

High-Intensity Treatment Arm, Interventions consisting of the mhGAP-IG interventions, activity scheduling and problem solving treatment.

MUAC, Mid-Upper Arm Circumference.

Level of significance set at p < 0.05.

With regard to infant nutrition, even though fewer (11.9%) adolescent mothers compared with adult mothers (16.6%) reported feeding their babies exclusively on breast milk for the first 6 months of life, the difference was not significant (p = 0.23). However, adolescent mothers had a higher rate of undernourished infants (defined as MUAC < 12.5 cm) compared with adult mothers, 14.8 and 6.3%, respectively (p = 0.008). The mean MUAC was significantly lower in the infants of adolescent mothers (p = 0.008), and this difference persisted even after we adjusted for their lower weight at birth (p = 0.04).

## Discussion

This study provides information regarding the rates of perinatal depression, its correlates, as well as postpartum differences between adolescents and adults who were enrolled into a randomized controlled trial of two forms of intervention for perinatal depression in a lower middle-income country. Results are presented comparing attitudes to motherhood, parenting skills, as well as growth and development of infants born to both groups of mothers. This study is unique in that we were able to compare the prevalence and outcomes of perinatal depression in a sample drawn from the same population who had undergone similar treatment exposures. While the severity of perinatal depression did not appear to differ between the adolescents and adults, the rate of depression was almost thrice that in adults. Overall, in the trial from which the current findings are drawn, treatment outcomes were similar in both the intervention and the control arms, with remission rates of 70 and 66%, respectively. We found the remission rates to be similar in the subsamples of adults and adolescents in the trial. However, the study was not powered for a comparison of subgroup treatment outcomes between the adolescent and adult mothers. The main differences between the adolescents and adults with perinatal depression in this study were in their attitudes and adjustments to pregnancy and motherhood, their parenting skills, and infant outcomes. In each of these, adolescent mothers had significantly lower scores than adult mothers and their infants.

There are wide variations in the reported prevalence of perinatal depression in adolescent populations ranging between 8 and 47%, probably reflecting methodological factors, especially differences in the ascertainment procedures and in the criterion definition of depression (9). Nevertheless, in this study and similar to reports in other populations of perinatal women from other parts of the world, the prevalence of depression in adolescents was much higher compared with that in adults (10–12).

We believe that our findings are an important contribution to the growing body of evidence that younger maternal age is a strong predictor of adverse pregnancy outcomes (32, 33). Compared with adult mothers, the adolescents delivered at an earlier gestational age and their infants had significantly lower birth weights than infants born to adult mothers. Relatedly, despite similar feeding patterns, the babies of adolescent mothers were less nourished at 6 months of life. Premature delivery and low birth weight infants are two important surrogate markers for adverse pregnancy outcome and infant mortality (33, 34). While studies have consistently found associations between infant birth weight and maternal age, the findings relating to the underlying risk factors for this association have not been consistent (4, 32). Associated factors for the shorter gestational age at delivery and lower birth weight of babies of adolescent mothers identified in the literature include social disadvantage, reduced antenatal visits, ethnicity, age at menarche, maternal height and net weight gain, and health behaviors during pregnancy as well as biological immaturity (33, 35, 36). Both the adult women and the adolescents in our study were recruited from essentially the same socioeconomic backgrounds and received similar antenatal care, suggesting that these two factors were not likely to explain the observed differences in this study. However, the poorer maternal attitudes observed in the adolescents during pregnancy could have resulted in reduced likelihood of adopting healthy eating and self-care habits that could have, in turn, impacted on fetal outcomes.

The results of this study suggest that while psychosocial interventions for depression seem to have been effective in reducing symptoms of depression among adolescent mothers, this remission of depression symptoms did not translate to improvement in parenting skills and adjustment to motherhood. The adolescents were less involved with and responsive to their infants as measured on the respective scales on the HOME inventory at 6 months, and the scores on the MAMA scales remained indicative of difficulties adjusting to parenting.

In this study, even though similar infant feeding patterns were reported by the adult and adolescent mothers, a significantly higher proportion of the infants of the adolescent mothers were undernourished as indicated by MUAC of less than 12.5 cm. The difference in the mean MUAC between adult and adolescent mothers persisted even after we controlled for the smaller birth weight of the infants of adolescent mothers. MUAC is a commonly used proxy for infant nutritional status and has been indicated as a more sensitive prognostic indicator for mortality than weight‐for‐height parameters in malnourished pediatric patients (37). Even though we did not collect data to enable us to determine the specific causes of the poorer infant nutrition in adolescent mothers, there is a possibility that this might be related to their poorer parenting skills, which could have affected infant feeding and the introduction of weaning diet.

The observations relating to worse perinatal outcomes in adolescent pregnancy are a relatively consistent finding from many parts of the world and have significant public health implications (4, 33). As noted in a recent report, there is a need for targeted interventions for this group of mothers especially in regions such as sub-Sahara Africa where high rates of adolescent pregnancy are still common. Interventions would need to target the prevention of child marriage and of unplanned pregnancies, provision of universal access to sexual and reproductive education (including contraception), and encouraging girls to receive secondary level education, which may serve to delay age at the birth of the first child (38). The findings of the current study suggest that for adolescents, approaches aimed at improving parenting skills would be a necessary component in developing care models and intervention packages for perinatal depression.

One major strength of this study is the selection of adult and adolescent mothers from the same population with similar treatment experience and socioeconomic backgrounds and, hence, reducing variations related to the quality of care. Our follow-up rates were high, and the study used standardized ascertainment procedures. Nevertheless, the study does have some limitations that should be considered in interpreting the findings. The first and more important is that the study was not powered to assess the effectiveness of treatment among adolescents with perinatal depression nor for subgroup comparison of outcome between adolescents and adults. Even so, previous studies exploring outcome of treatments among adolescents with perinatal depression have had fewer participants and employed less robust methodological approaches (16). Second, there are a number of unmeasured confounding factors that could have accounted for the higher rates of malnourished infants in the adolescent mothers, such as differences in weaning practices between adolescent and adult mothers. Another important limitation pertains to the assessment tools for the outcome measures (especially the HOME and MAMA); while these tools have been extensively used and validated in adult populations in other cultures, data regarding their psychometric properties and cultural acceptability in adolescent populations as well as in adults in low- and middle-income settings are not readily available. Notwithstanding these limitations, the findings of this study provide strongly suggestive evidence for a need to take into account the peculiar and unique characteristics of adolescent mothers with perinatal depression in designing an appropriate and effective intervention for the condition. This is particularly so given the salience of good parenting skills to adequate infant growth and development.

## Data Availability Statement

The datasets are available on request to the most senior author and PI of the study. E-mail:, ogureje@com.ui.edu.ng

## Ethics Statement

The EXPONATE study was carried out in accordance with the recommendations of the University of Ibadan/University College Hospital Ibadan Ethical Review Committee. Each individual participant provided written informed consent.

## Author Contributions

OG conceived the study and, along with BO, RA, PZ, and LK, obtained funding. BO produced the first draft of the manuscript and with input from OG, PZ, and RA. TB conducted the statistical analysis. All authors approved the final version.

## Funding

The EXPONATE study was funded by Grand Challenges Canada (0082–04).

## Conflict of Interest

The authors declare that the research was conducted in the absence of any commercial or financial relationships that could be construed as a potential conflict of interest.

The reviewer SH declared a shared affiliation, with no collaboration, with one of the authors BO to the handling editor.
